# Anti-Jo-1 autoantibodies: biomarkers of severity and evolution of the disease in antisynthetase syndrome

**DOI:** 10.1186/s13075-023-03116-5

**Published:** 2023-07-22

**Authors:** Robin Arcani, Louise Rey, Alice Mazziotto, Daniel Bertin, Gilles Kaplanski, Pierre-André Jarrot, Pierre Lafforgue, Geoffroy Venton, Xavier Heim, Patrick Villani, Jean-Louis Mège, Alexandre Brodovitch, Nathalie Bardin

**Affiliations:** 1 Internal Medicine and Therapeutics Department, CHU La Timone, Assistance Publique-Hôpitaux de Marseille (AP-HM), 264 Rue Saint-Pierre, 13005 Marseille, France; 2 Center for Cardiovascular and Nutrition Research (C2VN), INRA, 1260, INSERM UMR_S 1263, Aix-Marseille University, Marseille, France; 3 Biogénopôle, CHU La Timone, Assistance Publique-Hôpitaux de Marseille (AP-HM), Marseille, France; 4 Department of Internal Medicine and Clinical Immunology, CHU La Conception, Assistance Publique-Hôpitaux de Marseille (AP-HM), Marseille, France; 5 Department of Rheumatology, CHU Sainte-Marguerite, Assistance Publique-Hôpitaux de Marseille (AP-HM), Marseille, France; 6 Hematology and Cellular Therapy Department, CHU La Conception, Assistance Publique-Hôpitaux de Marseille (AP-HM), Marseille, France

**Keywords:** Anti-Jo-1 autoantibodies, Antisynthetase syndrome, Biomarkers

## Abstract

**Background:**

Anti-Jo-1 autoantibodies represent essential markers in the diagnosis of antisynthetase syndrome (ASS). In this retrospective study, we aimed to investigate whether their concentrations and fluctuations could both respectively reflect the severity and evolution of ASS.

**Methods:**

Between 2015 and 2020, clinical and biological features of ASS patients with at least one positive measure of anti-Jo-1 autoantibody were collected. At each serum sampling, we assessed myositis activity by using the Myositis Intention to Treat Activities Index (MITAX) and compared anti-Jo-1 concentrations with ASS severity, anti-Jo-1 concentrations between patients with and without active disease, and changes in anti-Jo-1 concentrations with disease activity.

**Results:**

Forty-eight patients with ASS had at least one positive determination of anti-Jo-1 concentration. Among them, twenty-nine patients had at least two determinations of anti-Jo-1 autoantibody in their follow-up. We showed that these autoantibody concentrations were significantly correlated with MITAX (*r* = 0.4, *p* = 0.03) and creatine kinase concentration (*r* = 0.34, *p* = 0.002) and that they were significantly higher in patients with active disease than in those with inactive disease (91.7 IU/L vs 44.4 IU/L, *p* = 0.016). During follow-up, we found a significant correlation between fluctuations of anti-Jo-1 autoantibody concentrations and MITAX score (*r* = 0.7, *p* < 0.0001).

**Conclusion:**

Our results suggest that anti-Jo-1 autoantibody concentration could be a predictive marker of the severity and evolution of ASS and show that their quantification could represent a precious tool for disease monitoring and for improving the therapeutic management of ASS patients.

## Introduction

Inflammatory myopathies are a broad spectrum of autoimmune diseases [1]. Subgroups of myositis were recently defined [2]: dermatomyositis, inclusion body myositis, immune-mediated necrotizing myopathy, and antisynthetase syndrome (ASS). ASS was individualized in patients with clinical manifestations and myositis-specific autoantibodies: antisynthetase autoantibodies. Anti-Jo-1 autoantibodies, directed against the histidyl-tRNA synthetase, are routinely detected in a quantitative way (ELISA or laser bead immunoassays) or in a qualitative/semi-quantitative way (immunoblots). They are the most commonly detected autoantibodies in patients’ sera who have ASS [3].

The disease status of ASS can be assessed with different clinical scorings, e.g., the Myositis Intention to Treat Activities Index (MITAX), which is an index part of the Myositis Disease Activity Assessment Tool (MDAAT) [4, 5]. However, in clinical practice, it is complex to assess disease severity and to monitor disease activity with the available tools. Moreover, there are multiple scores to assess disease activity, without any gold standard [6]. There is a trend towards an association between myositis activity and creatine kinase (CK) concentration, but it is an insufficient surrogate to assess disease severity [7, 8], so other biomarkers must be investigated. There are only a few studies assessing whether their concentration might be predictive of severity or useful monitoring markers for predicting the progression of ASS [9]. In this study, we evaluated if the quantification of anti-Jo-1 autoantibody concentration and its monitoring is associated with ASS severity and evolution, respectively.

## Materials and methods

### Patient

We conducted a retrospective study from the database of the Immunology Department of the University Hospitals of Marseille, France. We selected all patients with at least one positive measurement of anti-Jo-1 antibody between 2015 and 2020, with a probable or definite myositis diagnosis, based on Bohan and Peter’s criteria [10] and on the 2017 ACR/EULAR criteria for inflammatory myopathies [11]. Associated cancers were those declared from 3 years before to 3 years after ASS diagnosis [12].

### Disease activity measurement

Along with each serum sampling, we retrospectively evaluated myositis activity from the medical records. We retrospectively applied the MITAX [4]. The MITAX includes the assessment of the physician’s intention-to-treat active disease present during the preceding month in each of 7 organ systems (pulmonary, cardiovascular, muscular, constitutional symptoms, cutaneous, skeletal, and gastrointestinal) using a 4-point ordinal scale (0 points: no evidence or discount, 1 point: contentment, 3 points: beware, 9 points: active). To minimize the bias of a retrospective design, the MITAX was calculated by two independent physicians who experimented in myositis (RA and AM) blinded from the anti-Jo-1 antibody concentrations and the disease activity. The assessment of MITAX was used as a surrogate to estimate the disease severity at the time of anti-Jo1 measurement and to follow the activity of the disease over time (at each anti-Jo1 sampling). The anti-Jo-1 measurements were performed on the day of the clinical examination. A patient was classified with inactive disease when the following criteria were satisfied for 3 previous months: (1) no evidence of muscular, articular, cutaneous, or pulmonary activity; (2) serum CK concentration < 300 IU/L; (3) corticosteroid dosage < 10 mg/day with a stable dose of immunosuppressive therapy.

### Immunological tests

Antinuclear autoantibodies (ANA) were detected by indirect immunofluorescence on HEp-2 cells (Bio-Rad Laboratories, Hercules, CA) at a screening dilution of 1/160. Anti-extractable nuclear antigen (ENA) antibodies including anti-SSA/Ro 60 kDa, anti-SSB/La, anti-Ro52kDa/TRIM21, anti-RNP, anti-Sm, anti-Scl70, anti-centromere B, and anti-Jo-1 autoantibodies were quantified using commercially available EliA™ kits (Phadia, Uppsala, Sweden; now part of Thermo Fisher Scientific, Waltham, MA). Cutoff values for anti-ENA were 10 IU/m. The sera were tested for anti-Jo-1, anti-PL7, anti-PL12, anti-EJ, anti-OJ, anti-MDA5, anti-Mi2, anti-TIF1γ, anti-SRP, anti-SAE1, anti-SAE2, and anti-NXP2 autoantibodies with BlueDiver® Myositis Profile 12-OJ immunodots (Alphadia SA/SN, Wavre, Belgium) when prescribed by the physicians. We assessed the changes in anti-Jo-1 concentration according to the result of the EliA™ kits.

### Ethical

This study was approved by the Institutional Review Board of Assistance Publique-Hôpitaux de Marseille (GDPR number PADS21-270). The study was conducted according to the Declaration of Helsinki.

### Statistical analysis

Quantitative variables were described using means and standard deviations (SD): categorical variables using numbers and percentages. Quantitative data were compared using Student’s *t* or Mann–Whitney *U* tests, while qualitative data were compared with the chi-square or Fisher’s exact test when appropriate. Spearman’s rank correlations were calculated to quantify the relationships between disease activity and anti-Jo-1 antibody concentrations. Some relationships between disease activity and anti-Jo-1 antibody concentrations were calculated considering MITAX subscorings as ordinal variables using the Kruskall-Wallis and Dunn’s multiple comparison tests. We defined correlations as follows: no correlation, weak, moderate, or strong correlations when *r* was < 0.25 0.25–0.5, 0.5–0.75, and > 0.75, respectively. Tests were two-sided, with *p*-values < 0.05 considered to be significant. All analyses were performed with the R software (R Foundation for Statistical Computing, Vienna, Austria) and GraphPad Prism 8.0.0 (GraphPad Software, San Diego, USA). The study was conducted in accordance with the recommendations of the STROBE initiative.

## Results

### Description of the cohort (Table 1)

**Table 1:** Demographic and clinical features of the patients

Characteristics	Whole cohort (*n* = 48)	Patients with one unique sampling of anti-Jo-1 (*n* = 19)	Patients with at least 2 samplings of anti-Jo-1 (*n* = 29)	*p*-value
Age at diagnosis^a^	53.9 ± 14.8	54.6 ± 15.8	53.5 ± 14.5	0.82
Female/male	35/13	12/7	23/6	0.22
Duration of follow-up in months^a^	93.6 ± 89.3	94.7 ± 106.8	93 ± 78.6	0.95
Clinical feature^b^				
Myositis	39 (81.3)	15 (78.9)	24 (82.8)	0.74
Arthralgia/arthritis	39 (81.3)	16 (84.2)	23 (79.3)	0.67
Interstitial lung disease	35 (72.9)	12 (63.2)	23 (79.3)	0.22
Mechanic’s hands	22 (45.8)	6 (31.6)	16 (55.2)	0.11
Raynaud’s phenomenon	10 (20.8)	3 (15.8)	7 (24.1)	0.49
Rash	3 (6.3)	1 (5.3)	2 (6.9)	0.82
Digestive symptoms	12 (25)	1 (5.3)	11 (37.9)	0.011
**Treatment lines** ^**b**^				
Corticosteroids	45 (93.8)	18 (94.7)	27 (93.1)	0.82
Methotrexate	21 (43.8)	8 (42.1)	13 (44.8)	0.85
Azathioprine	15 (31.3)	5 (26.3)	10 (34.5)	0.55
Rituximab	11 (22.9)	3 (15.8)	8 (27.6)	0.34
Intravenous immunoglobulins	9 (18.8)	5 (26.3)	4 (13.8)	0.28
Mycophenolate mofetil	7 (14.6)	1 (5.3)	6 (20.7)	0.14
Cyclophosphamide	4 (8.3)	1 (5.3)	3 (10.3)	0.54
Cyclosporin A	1 (2.1)	0 (0)	1 (3.4)	0.41
Number of treatment lines^a^	2.4 ± 1.2	2.2 ± 1	2.5 ± 1.3	0.29

^a^Mean ± SD

^b^*n* (%)

Between 2015 and 2020, 48 patients (35 female patients, 72.9%) with at least one positivity of anti-Jo-1 antibody were retrospectively included. All patients had a probable or definite inflammatory myopathy according to Bohan and Peter’s criteria [10] and to the 2017 ACR/EULAR criteria for inflammatory myopathies [11]. The mean age at the beginning of symptoms was 52.1 ± 14.4 years, and the mean age at diagnosis of ASS was 53.9 ± 14.8 years. The mean follow-up was 93.6 ± 89.3 months (range: 9–372). There was an associated autoimmune disease in 10 patients (20.8%): rheumatoid arthritis (RA) (*n* = 6, 12.5%), primary Sjögren’s syndrome (*n* = 2, 4.2%), systemic lupus erythematosus (SLE) (*n* = 2, 4.2%), Hashimoto’s thyroiditis (*n* = 1, 2.1%), systemic sclerosis (*n* = 1, 2.1%), and antiphospholipid syndrome (*n* = 1, 2.1%). There was an associated neoplasm in 3 patients (6.3%): 2 colic adenocarcinomas and 1 breast cancer. CK concentration was elevated (> 170 IU/L) at least at one antibody measurement in 14/44 patients (31.8%) during follow-up. Four patients were under statins during at least one point of the follow-up (atorvastatin *n* = 3, simvastatin *n* = 1). Twenty-seven patients (56.3%) were negative for ANA. Besides positivity for anti-Jo-1, screening for other autoantibodies showed anti-ENA to be negative in 25 patients (52.1%). Sixteen patients (33.3%) presented an anti-Ro52kDa/TRIM21 antibody, 5 (10.4%) an anti-SSA 60 kDa antibody, 4 (8.3%) an anti-centromere B, and 2 (4.2%) an anti-SSB antibody. All patients tested (29/29) were positive for anti-Jo-1 screening with immunodot assay. Two patients (6.9%) of 29 screened for myositis-specific or myositis-associated autoantibodies were positive for anti-PL-12 and anti-HMGCR, respectively. Six patients underwent muscular biopsy: in 5, typical patterns of inflammatory myopathy were found. All patients received treatment for ASS: 13 (27.1%), 14 (29.2%), 15 (31.3%), and 6 (12.5%) patients received one, two, three, or more than 3 lines of treatment, respectively. Corticosteroids were the most often used treatment (45 patients, 93.8%). Of 48 patients, 29 had at least two determinations of anti-Jo-1 antibody concentration. In these monitored patients, there was a mean of 3.2 ± 2.1 determinations of anti-Jo-1 antibody concentration per patient. Sixteen patients (55.2%) had 2 determinations of anti-Jo-1 concentration and 8 patients had at least 4 determinations (27.6%) of anti-Jo-1 concentration.

### Anti-Jo-1 concentration is a good marker of ASS severity in contrast to CK concentration

The severity of ASS, assessed by CK concentration, MITAX (total scoring and subscorings in each organ system), and disease activity, was analyzed according to the anti-Jo-1 concentrations.

**Fig. 1:**
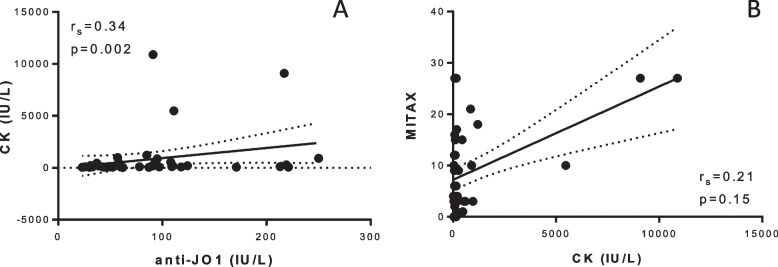
Correlation between CK concentration and anti-Jo-1 concentration (**A**) and MITAX (**B**)

In the whole cohort of 48 patients with at least one measurement of anti-Jo-1 autoantibody concentration, we found a significant weak correlation between anti-Jo-1 and CK concentration (*r* = 0.34, CI95% 0.044–0.59, *p* = 0.002, Fig. 1A), whereas no correlation was found between CK concentration and disease activity as evaluated by MITAX (*r* = 0.21, CI95%: − 0.094–0.49, *p* = 0.15, Fig. 1B).

**Fig. 2:**
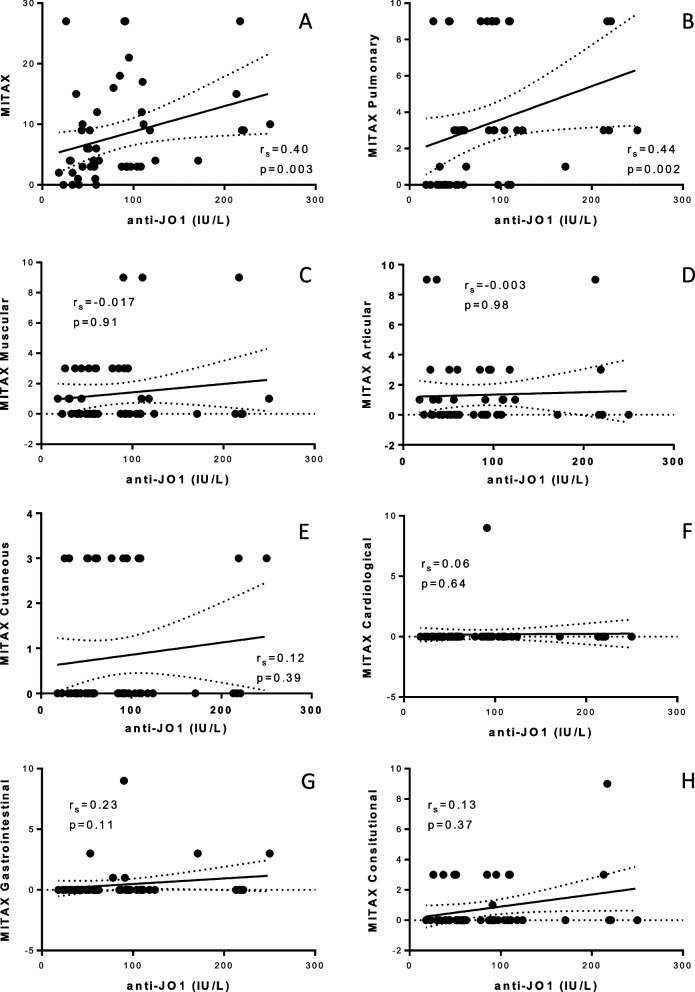
Correlation between anti-Jo-1 concentration and global (**A**), pulmonary (**B**), muscular (**C**), articular (**D**), cutaneous (**E**), cardiological (**F**), gastrointestinal (**G**), and constitutional (**H**) disease activity

In addition, we found a significant, but weak, association between anti-Jo-1 concentrations and MITAX (*r* = 0.4, CI95%: 0.13–0.62 *p* = 0.003, Fig. 2A). There was a significant association between anti-Jo-1 concentration and pulmonary MITAX (*r* = 0.44, *p* = 0.002, mean anti-Jo-1 concentration: 50.5 ± 28.4 vs 108.9 ± 66.2, *p* = 0.0062 between patients with MITAX: 0 vs MITAX: 3, Fig. 2B). We did not find any statistical association between anti-Jo-1 concentration and the muscular, articular, cutaneous, cardiological, gastrointestinal, or constitutional disease activity.

**Fig. 3:**
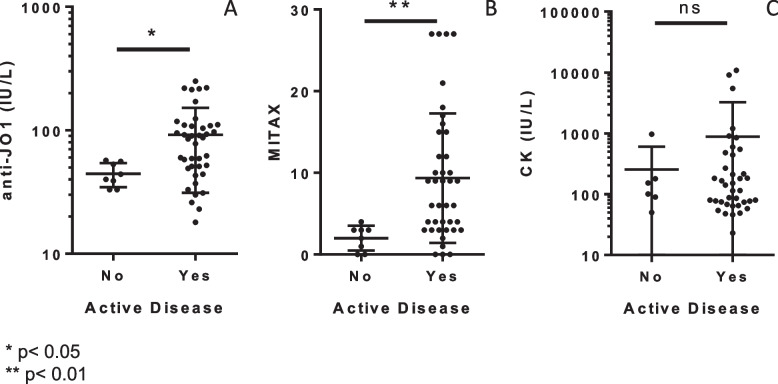
Correlation between disease activity and anti-Jo-1 concentration (**A**), MITAX (**B**), and CK concentration (**C**)

When comparing anti-Jo-1 concentrations to patients with active and inactive disease, it showed that anti-Jo-1 concentrations were higher in those with active disease (mean anti-Jo-1 concentration: 91.7 IU/L vs 44.4 IU/L, *p* = 0.016, Fig. 3A). Similar results were obtained with MITAX (9.7 vs 2, *p* < 0.01, Fig. 3B), in contrast to CK concentration for which no significant difference was observed between patients (with active or inactive disease) (882 IU/L vs 257 IU/L, *p* > 0.05, Fig. 3C).

### Anti-Jo-1 concentrations represent a follow-up marker in ASS evolution

**Fig. 4:**
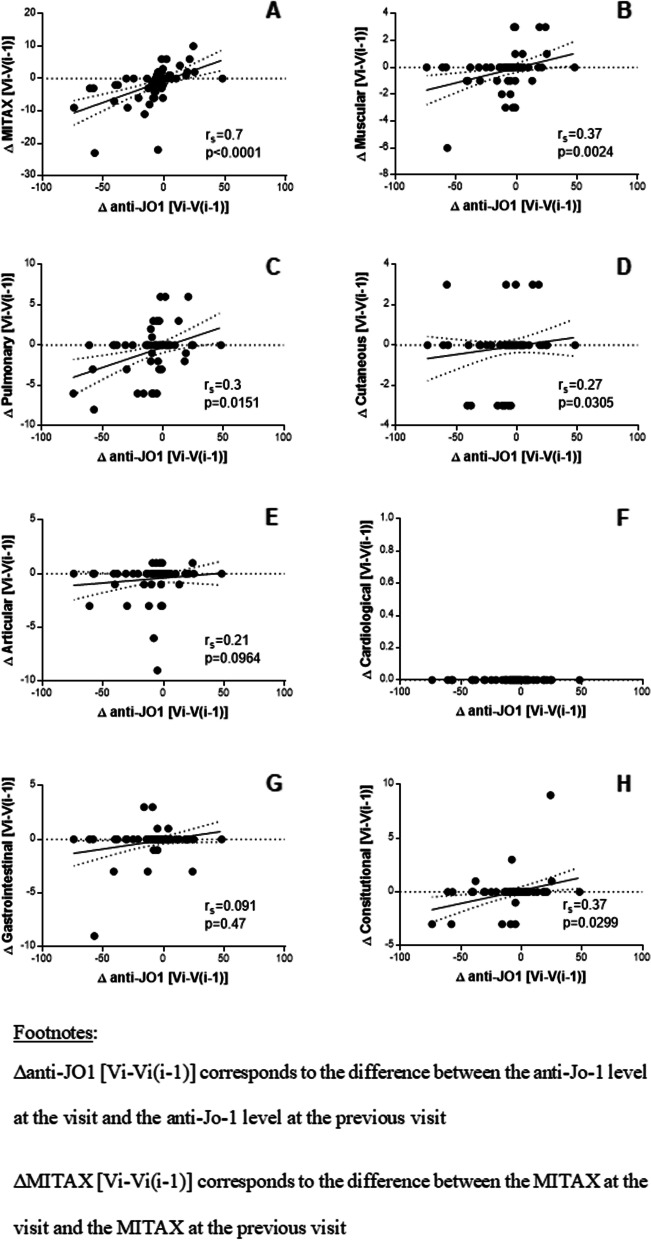
Correlation between anti-Jo-1 concentration changes and disease activity evolution (global (**A**), muscular (**B**), pulmonary (**C**), cutaneous (**D**), articular (**E**), cardiological (**F**), gastrointestinal (**G**), constitutional (**H**) disease activity) in each patient (*n* = 29) during the follow-up

In 29 patients with at least two measurements of anti-Jo-1, followed for 93 months on average, we investigated at each visit a potential association between the changes of anti-Jo-1 concentrations and those of MITAX, including subscorings in each organ system. As presented in Fig. 4, fluctuations of anti-Jo-1 concentrations were significantly and moderately correlated with those of MITAX (*r* = 0.7, CI95%: 0.55–0.81, *p* < 0.0001, Fig. 4A). Similarly, the anti-Jo-1 concentration variations were also weakly correlated with those of muscular (*r* = 0.37, CI95%: 0.13–0.57, *p* = 0.0024, Fig. 4B), pulmonary (*r* = 0.3, CI95%: 0.053–0.51, *p* = 0.015, Fig. 4C), and cutaneous (*r* = 0.27, CI95%: 0.019–0.49, *p* = 0.03, Fig. 4D) MITAX subscorings. We did not find any statistical association between anti-Jo-1 concentration changes and CK concentration changes (*r* = 0.28, CI95%: − 0.098–0.59, *p* = 0.13) or between CK concentration changes and MITAX changes (*r* =  − 0.05, CI95%: − 0.64–0.58, *p* = 0.86).

## Discussion

Predicting ASS progression presents a crucial interest to adapt therapeutic management. Our results showed that quantification of anti-Jo-1 autoantibody concentration is of interest because it is associated with disease severity, and their monitoring could be useful to monitor ASS evolution. These autoantibodies are routinely searched and quantified in medical laboratories, making them available and reliable biomarkers in clinical practice.

In the literature, numerous data evidenced the major role of anti-Jo-1 autoantibodies as the most prevalent diagnostic marker for ASS patients [3], but few data investigated their potential usefulness in disease monitoring. In agreement with our results, Stone et al. showed a correlation between anti-Jo-1 concentrations and the global severity of the disease and between anti-Jo-1 concentration and muscular and articular activities [13]. They also reported 11 patients with several determinations of anti-Jo-1 concentration and concomitant clinical assessment based on MITAX evaluation, in which a correlation was found between anti-Jo-1 fluctuations and changes in ASS activity. Our study, conducted on 29 patients with at least two measurements of anti-Jo-1 concentration, represents the largest cohort of followed patients with this rare autoimmune disease in the literature. As such, Aggarwal et al. [14] found that clinical improvement post-rituximab was associated with a decrease in anti-Jo-1 concentration in a cohort of patients treated with rituximab for inflammatory myopathies. These data suggest that quantification of anti-Jo-1 autoantibodies can help in identifying patients with poor outcomes, who require more aggressive treatment.

These findings indicate that anti-Jo-1 autoantibodies, myositis-specific autoantibodies, could be involved in the pathogenicity of myositis as well as in the flare of the disease. Tissue modifications of the expression of the antigenic target, histidyl-tRNA-synthetase, may be responsible for the recruitment and activation of both innate and adaptive immune cells. Therefore, anti-Jo-1 autoantibodies, reflecting autoimmunization, could be correlated with the cellular response [15]. Some studies showed that anti-Jo-1 autoantibodies were found when T lymphocytes (against Jo-1) were present [16]. Moreover, the B-cells’ expansion, and consequently the autoantibodies production, is amplified by the TCD4 cell response [17]. Taken together, our results and the beneficial effect of B-cells’ depletion in myositis [14, 18] are an element for the role of anti-Jo-1 autoantibodies in the emergence of tissular lesions in myositis.

Our study had some biases. Our sample size was quite small. This is common when studying rare diseases like ASS. However, compared to the data reported in the literature, our cohort is one of the largest. One of the limitations of our study is due to its retrospective design. However, our results, in agreement with previous studies [13, 14], pave the way to a multicenter prospective study.

In conclusion, like the monitoring of anti-dsDNA in SLE or of C-reactive protein in RA [19, 20], the quantification of anti-Jo-1 autoantibodies represents an attractive biomarker to monitor the disease activity. Managing ASS is a major challenge, and there are no consensual guidelines to address this issue. The anti-Jo-1 antibody quantification can be another tool to assess the evolution of the disease. We could suggest their quantification at each clinical assessment to help the practitioners’ decisions. Multicenter prospective studies will be necessary to validate our findings and to assess the timing and frequency of quantification of anti-Jo-1 autoantibodies according to the therapeutic strategies applied.

## Data Availability

The data that support the findings of this study are available from the corresponding author, but restrictions apply to the availability of these data and so are not publicly available. Data are however available from the authors upon reasonable request and with permission from the corresponding author.
